# Ammonium Impacts Methane Oxidation and Methanotrophic Community in Freshwater Sediment

**DOI:** 10.3389/fbioe.2020.00250

**Published:** 2020-03-31

**Authors:** Yuyin Yang, Tianli Tong, Jianfei Chen, Yong Liu, Shuguang Xie

**Affiliations:** ^1^State Key Joint Laboratory of Environmental Simulation and Pollution Control, College of Environmental Sciences and Engineering, Peking University, Beijing, China; ^2^Key Laboratory of Water and Sediment Sciences (Ministry of Education), College of Environmental Sciences and Engineering, Peking University, Beijing, China

**Keywords:** ammonium, freshwater lake, methane oxidation, methanotroph, *pmoA* gene, *pmoA* transcripts

## Abstract

Lacustrine ecosystems are regarded as one of the important natural sources of greenhouse gas methane. Aerobic methane oxidation, carried out by methane-oxidizing bacteria, is a key process regulating methane emission. And ammonium is believed to greatly influence aerobic methane oxidation activity. To date, disagreement exists in the threshold of ammonium effect. Moreover, knowledge about how aerobic methanotrophic community composition and functional gene transcription respond to ammonium is still lacking. In the present study, microcosms with freshwater lake sediment were constructed to explore the effect of ammonium level on aerobic methanotrophs. Methane oxidation potential, and the density, diversity and composition of *pmoA* gene and its transcripts were examined during 2-week incubation. A negative impact of ammonium on aerobic methane oxidation potential and a positive impact on *pmoA* gene density were observed only at a very high level of ammonium. However, *pmoA* gene transcription increased notably at all ammonium levels. The composition of functional *pmoA* gene and transcripts were also influenced by ammonium. But a great shift was only observed in *pmoA* transcripts at the highest ammonium level.

## Introduction

Methane, a critical greenhouse gas, is one of the major products of carbon metabolism in freshwater lake (Bastviken et al., 2004). Aerobic methane oxidation performed by methane-oxidizing bacteria (MOB) is a major pathway to reduce methane emission (Fergala et al., 2018). Up to 30–99% of the total methane formed in anoxic sediment environment can be oxidized by methanotrophs (Bastviken et al., 2008). Therefore, aerobic methane oxidation is a critical biochemical process in freshwater lake. This process can be greatly mediated by the environmental changes (e.g., eutrophication) induced by anthropogenic activities (Borrel et al., 2011).

The increasing nutrient input into freshwater lakes has greatly raised the availability of dissolved organic carbon (DOC) as well as nitrogen and phosphorus, which exerts a profound impact on methane oxidation (Liikanen and Martikainen, 2003; Veraart et al., 2015). Among various types of nutrients, ammonium, an essential compound in nitrogen cycling, has attracted great attention. Methane and ammonium share similar chemical structure, and ammonium can compete with methane for the binding site of methane monooxygenase, a key enzyme in methane oxidation (Bédard and Knowles, 1989). Excess ammonium can also lead to the competition between methane oxidizers and ammonium oxidizers for oxygen. On the other hand, with high oxygen availability or low *in situ* nitrogen content, methane oxidation can also be stimulated by ammonium addition (Rudd et al., 1976). Besides, ammonium might also induce differential expression of pMMO encoding genes (Dam et al., 2014). Hence, the effects of ammonium on methane oxidation in natural ecosystems are complex (Bodelier and Laanbroek, 2004), and previous studies have documented contradictory results, such as inhibition (Bosse et al., 1993; Nold et al., 1999; Murase and Sugimoto, 2005), no effect (Liikanen and Martikainen, 2003), or stimulation (Rudd et al., 1976; Bodelier et al., 2000). The effect of ammonium on methane oxidation might largely depend on the characteristics of the studied ecosystem and *in situ* environment (Bodelier and Laanbroek, 2004; Borrel et al., 2011).

Previous studies about the ammonium effect on methane oxidation in freshwater lake mainly focused on either oxidation rate or net methane flux (Bosse et al., 1993; Liikanen and Martikainen, 2003; Murase and Sugimoto, 2005), while MOB community dynamics has attracted little attention. MOB play a fundamental role in regulating methane emission from freshwater sediment (Bastviken et al., 2008). The abundance, transcription, and community structure of MOB may also be affected by the extra ammonium input (Shrestha et al., 2010). The difference of MOB community structures may further lead to various responses of methane oxidation to nitrogen level (Mohanty et al., 2006; Nyerges and Stein, 2009; Jang et al., 2011). Therefore, identification of the variation of MOB community can be helpful to understand how ammonium input influences methane oxidation. MOB community change under ammonium stress has been observed in various soils, such as agriculture soil (Seghers et al., 2003; Shrestha et al., 2010) and landfill soil (Zhang et al., 2014). The results of these previous studies suggested that the effect of ammonium on MOB community might be habitat-related. Field work results did suggest that *in situ* ammonium concentration might be a crucial factor regulating the structure of MOB community in freshwater sediment (Yang et al., 2016). A direct evidence for the influence of ammonium on MOB community in freshwater lake sediment is still lacking. Little is known about the transcription change of *pmoA* gene under ammonium pressure.

A number of freshwater lakes in China are suffering from eutrophication. The MOB communities in these ecosystems have been under high ammonium pressure, and were of a great importance in regulating methane emission from these lakes. In the present study, we constructed microcosms with eutrophic freshwater lake sediment to investigate the MOB community shift at different ammonium dosages. The main objective was to demonstrate how ammonium regulated methane oxidation activity via MOB community composition and functional gene transcription in lake sediment.

## Materials and Methods

### Sediment Characteristics

Dianchi Lake is a large shallow freshwater lake (with total surface area of 309 km^2^ and average water depth of 4.4 m) in southeast China (Yang et al., 2016), and it is suffering from anthropogenically accelerated eutrophication (Huang et al., 2017). Surface sediment (0–5 cm) (24.9286N, 102.6582E, Supplementary Figure S1) was obtained from the north part of Dianchi Lake in October, 2017. Overlying water at the same site was collected with a plexiglass water sampler. And pH and water dissolved oxygen (DO) of overlying water were immediately measured with electrode sensors. For measurement of other physicochemical properties, sediment sample was transported to laboratory at −20°C and then analyzed according to the literature (Wang, 2012). About 50 mL of sediment sample was centrifuged at 5,000 rpm for 10 min, and the supernatant was used to determine the concentration of ammonium nitrogen in pore water. Another 1 L of surface sediment was immediately transported to laboratory at 4°C for incubation experiment. Physico-chemical characteristics of environmental sample could be found in Supplementary Table S1.

### Experimental Setup

Sediment was placed at room temperature for 24 h for the recovery of microbes. Then the sediment sample was well-mixed to ensure the parallelism in subsequent incubation experiments. A series of 50-mL autoclaved serum bottles (as microcosms) were added with 10 mL of sediment aliquot (containing about 1 g dry sediment). A total of 111 microcosms were constructed. Three of these microcosms were autoclaved before incubation, to verify that the methane consumption was attributed to the activity of microorganisms. Among the rest of microcosms, a total of six different treatments (A–F) were established. Treatments B–F contained 1 mL of NH_4_Cl at the levels of 5, 20, 50, 100, and 200 mM, respectively, while treatment A was amended with 1 mL diluted water as the blank control. For each treatment, 18 microcosms were constructed, including half used for molecular analyses and another half for MOP measurement. These microcosms were closed with butyl rubber stoppers and incubated for 14 days at 25°C at 100 rpm in dark.

At each sampling time point [day 1 (12 h after incubation), day 7 or day 14], triplicate samples were sacrificed for physicochemical measurement and molecular analysis.

Sediment sample from each serum bottle was transferred into a Falcon tube, and then centrifuged at 5000 rpm for 10 min. The supernatant was filtered with a 0.2-μm syringe filter, and its ammonium level was measured using Nessler reagent-colorimetry. The rest of the replicated sediment samples were pooled and immediately used for nucleic acid extraction. Methane oxidation potential (MOP) measurement was carried out at the same time points at described above. Another three bottles of each treatment were opened and shaken to provide ambient air, then closed again with butyl rubber stoppers. 1 mL of headspace air was replaced by CH_4_ (99.99%) with an air-tight syringe, resulting in a final concentration of approximately 1.6% CH_4_ in headspace. Samples were shaken vigorously to mix, and incubated in dark at 25°C, 100 rpm for 24 h. After incubation, 0.1 mL of headspace gas was sampled and measured using a GC126 gas chromatograph. Autoclaved control was also processed to exclude methane loss due to dissolution or airtightness.

### Nucleic Acid Extraction, Reverse Transcription and Quantification

Sediment DNA and RNA were extracted with PowerSoil DNA Isolation Kit and PowerSoil Total RNA Isolation Kit (MoBio, United States), respectively, and were evaluated with Nanodrop 2000 (Thermo Fisher Scientific, United States). RNA further was prepared at a similar concentration before further analysis. Real-time PCR of *pmoA* gene was performed on a CFX Connect cycler (Bio-Rad, United States), using the primers A189f/mb661r following the PCR conditions reported by Liu et al. (2015). Reactions were proceeded using TransStart Top Green qPCR Kit (Transgen Biotech Co., Ltd, China). Gene transcripts were quantified in a one-step RT-qPCR using a TransScript Green One-step qRT-PCR Kit (Transgen Biotech Co., Ltd, China). Possible gDNA contamination was removed with DNase before reverse transcription. Melting curve analyses were carried out at the end of PCR run to verify the amplification specificity. Each measurement was carried out with three technical replicates, and result was given in average ± standard deviation. Standard curve was constructed with serially diluted plasmids containing *pmoA* gene fragment, and the efficiency and *r*-square were 91.5% and 0.998, respectively.

### Terminal Restriction Fragment Length Polymorphism (T-RFLP) Fingerprinting

TRFLP fingerprinting was carried out both on DNA and RNA samples. DNA *pmoA* gene fragment was amplified with the primers A189f/mb661r, with the forward primer A189f modified with FAM at 5′-end. PCR reactions were performed as previously described (Liu et al., 2015). RNA samples were reversely transcripted into cDNA with *pmoA* gene specific primer and then amplified following the same protocol with DNA samples. Reverse transcription was carried out using One-step gDNA removal and cDNA synthesis kit (Transgen Biotech Co., Ltd, China). The 20-μL reaction solution contained 1 μL EasyScript RT/RI Enzyme Mix, 1 μL gDNA remover, 10 μL 2 × ES Reaction Mix, 2 pmol of gene specific primers, and 1 μL RNA template. The reaction mixture was incubated at 42°C for 30 min, and the enzymes were deactivated at 85°C for 5 s. Due to the FAM modification of PCR primer A189f, the PCR products of DNA and cDNA were fluorescently labeled at 5′-end.

The PCR products were purified using TIANquick Mini Purification Kit (TIANGEN Bitotech Co., Ltd, China). Approximately 20 ng purified amplicons were digested with restriction endonuclease *BciT130* I (Takara Bio Inc., Japan) following the conditions recommended by the manufacturer’s instruction. Electrophoresis of digested amplicons was fulfilled by Sangon Biotech (China) using an ABI 3730 DNA analyzer (Thermo Fisher Scientific, United States). The length of T-RFs was determined by comparing with internal standard using GeneScan software. T-RFs with similar length [less than 2 base pairs (bp) difference] were merged, and T-RFs shorter than 50 bp or longer than 508 bp were excluded from the dataset. The ratio of the peak area of a given T-RF to the total area was assigned as its relative abundance. Minor T-RFs (with the proportion of less than 0.5%) were excluded from further analysis. Each of the remained T-RFs was regarded as an OTU (Operational Taxonomic Unit) in the subsequent analysis. Shannon diversity indices of *pmoA* gene and transcripts were calculated based on DNA and RNA T-RFs, respectively.

### Cloning, Sequencing and Phylogenetic Analysis

*pmoA* gene clone library was generated with the mixed DNA PCR products using a TA cloning kit (TransGen Biotech Co., Ltd, China). Randomly picked clones were subjected to sequencing. A total of 93 *pmoA* sequences were retrieved and the corresponding T-RFs when digested with endonuclease *BciT130* I were predicted using the online software Restriction Mapper^1^. Several sequences of each T-RF, together with their reference sequences from the GenBank database, were used to construct a neighbor-joining phylogenetic tree with MEGA 7 (Kumar et al., 2016). And a taxonomic unit could be assigned to each T-RF accordingly. The tree was visualized with online software iTOL (Letunic and Bork, 2016). The *pmoA* sequences used in phylogenetic analysis were deposited in GenBank database, and the accessions were shown in Figure 3. The phylogenetic tree linked T-RFs to certain phylogenetically distinct groups, thus making it possible to demonstrate the composition of MOB community and *pmoA* transcripts based on T-RFLP profiling.

### Statistical Analysis

Two-way ANOVA (analysis of variance) was used to determine the effects of ammonium concentration and incubation time, as well as their interaction effect on methane oxidation potential, gene abundance and transcription. Ammonium addition and incubation time were regarded as two fixed factors. One-way ANOVA and Student–Newman–Keuls *post hoc* test was adopted to detect the difference among treatments. These analyses were carried out in *R*, using *R* packages stats (version 3.4.4) and agricolae (version 1.2-8).

Clustering analysis was adopted based on Bray–Curtis dissimilarity, in order to demonstrate the similarity between MOB community and transcription at different time points during incubation. Redundancy Analysis (RDA) was applied as a multivariance regression tool to demonstrate how much community profiling could be explained by ammonium amendment and incubation. Significance was tested based on permutation test. Both clustering analysis and RDA were carried out in R with R package Vegan (version 2.4-6) (Oksanen et al., 2018).

## Results

### Methane Oxidation Potential

**FIGURE 1:**
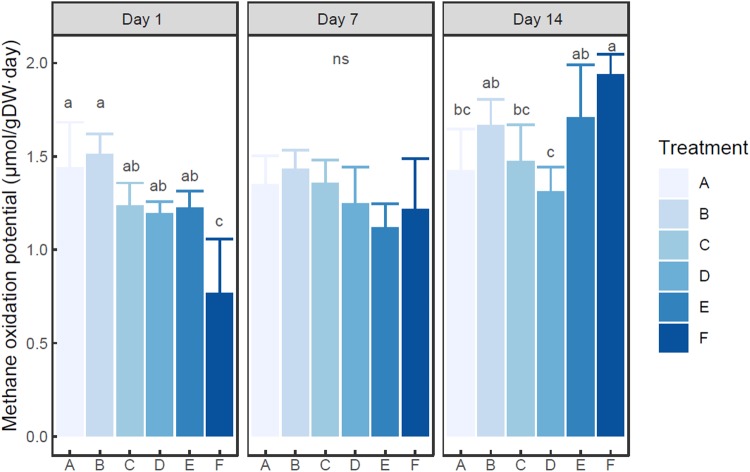
Change of methane oxidation potential in the microcosms with different treatments. Error bar indicates standard deviation (*n* = 3). Asterisk indicates the significance between experiment group and control group (*P* < 0.05). ‘ns’ indicates no significant difference among treatments at a given time.

Ammonium was found to quickly deplete in each ammonium added microcosm (Supplementary Figure S2). MOP varied from 0.77 (in the microcosm F with 200 mM ammonium on day 1) to 1.94 (in the microcosm F with 200 mM ammonium on day 14) μmol/g dry sediment day (Figure 1), while autoclaved control did not show notable methane oxidation (data not shown). Based on two-way ANOVA, both ammonium concentration (treatment) and incubation time had significant effects on MOP (*P* < 0.01), and their interaction was also significant (*P* < 0.05). The MOP in the microcosm with treatment A (with no external ammonium addition) did not show a significant difference among incubation times (*P* > 0.05). Based on *post hoc* test (Figure 1), at each time point, the microcosm with treatment B (5 mM ammonium) had slightly higher MOP than the microcosm with control group (A). At days 1 and 7, the microcosms with 20–100 mM ammonium addition had slightly lower MOP than the un-amended microcosm. However, at each time, no statistical difference in MOP was observed among the microcosms with treatment A-E (0–100 mM ammonium addition). Moreover, the microcosm with treatment F (200 mM ammonium) tended to have significantly lower MOP than other microcosms on day 1 (*P* < 0.05), but significantly higher MOP on day 14 (*P* < 0.05). On day 7, no statistical difference in MOP was found between the microcosm with treatment F and any other microcosms.

### Densities of *pmoA* Gene and Transcripts

**FIGURE 2:**
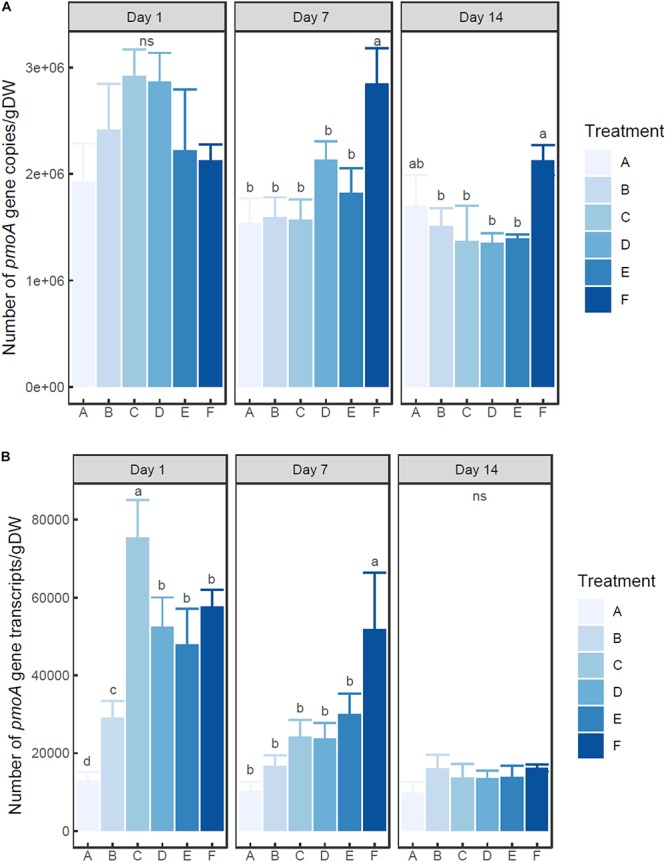
Changes of *pmoA* gene **(A)** and transcript **(B)** abundance in the microcosms with different treatments. Error bar indicates standard deviation (*n* = 3). Asterisk indicates the significance between experiment group and control group (*P* < 0.05). ‘ns’ indicates no significant difference among treatments at a given time.

Two-way ANOVA indicated that the number of both *pmoA* gene and transcripts was significantly influenced by ammonium concentration and incubation time (*P* < 0.01) (Figures 2A,B). The abundance of *pmoA* gene in the control group (A) showed no significant difference among times (0.05 < *P* < 0.1). On day 1, the microcosms with treatment C and D (20–100 mM ammonium) had higher (but not significant) *pmoA* gene abundance than other microcosms. However, at days 7 and 14, the microcosm with treatment F (with the highest ammonium addition of 200 mM) had the highest *pmoA* gene abundance.

**FIGURE 3:**
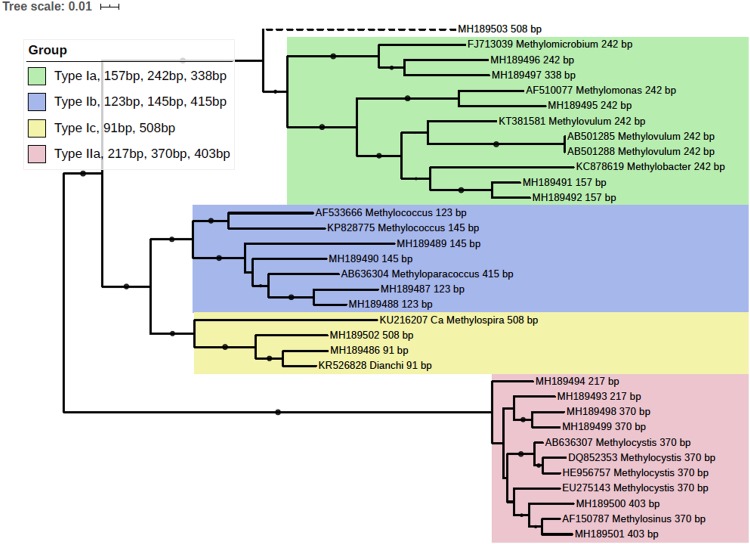
Phylogenetic tree of obtained *pmoA* sequences and reference sequences from the GenBank database. The predicted cut sites were shown after the accession numbers of sequences. The dots at branches represent the support values from bootstrap test. Branch support values of no less than 50 were dotted. The bar represents 1% sequence divergence based on neighbor-joining algorithm.

At each time point, *pmoA* transcripts in the un-amended microcosm was less abundant than those in amended microcosms. On day 1, the highest number of transcripts was observed in the microcosm with treatment C (20 mM), followed by the microcosms with treatments D, E, and F (50–200 mM). The microcosm with treatment B (5 mM) had much lower *pmoA* transcript abundance than other ammonium added microcosms (*P* < 0.05) (Supplementary Table S2). On day 7, *pmoA* transcript abundance tended to increase with the level of added ammonium, although statistical difference in *pmoA* transcript abundance was only observed between treatment F and other treatments. On day 14, no significant difference in *pmoA* transcript abundance was detected among treatments (*P* > 0.05).

The ratio of transcripts to *pmoA* gene varied with ammonium concentration and incubation time (Supplementary Figure S3). The ratio tended to decrease with time in ammonium amended microcosms. Moreover, at days 1 and 7, the ratio tended to increase with the increasing ammonium concentration.

### T-RFLP Fingerprinting

*In silico* analysis of the cloned *pmoA* sequences showed that restriction enzyme *BciT130* I could well capture *pmoA* gene diversity and present a good resolution among different subgroups of aerobic methanotrophs (see Supplementary Figure S4 and Supplementary Discussion). Most of the T-RFs retrieved in the current study could be assigned to certain MOB groups, while some of the T-RFs from *pmoA* transcripts could not match the cut site predicted from the sequences in clone library. The obtained *pmoA* sequences could be grouped into four clusters (Figure 3), which could be convincingly affiliated with known methanotrophic organisms. Three clusters were affiliated with Type I MOB (*Gammaproteobacteria*), which could be further divided into several subgroups. Cluster 1 contained 157, 242, and 338 bp T-RFs that could be related to Type Ia MOB, the most frequently detected methanotrophs in freshwater lakes (Borrel et al., 2011). The 157 bp and 338 bp T-RFs might be affiliated with *Methylobacter* and *Methylomicrobium*, respectively. However, the 242 bp T-RF could not be convincingly assigned to a certain genus because of the highly similar *pmoA* sequences of Type Ia organisms. Cluster 2 was composed of three different T-RFs, and could be affiliated with *Methylococcus* and *Methyloparacoccus*. Cluster 3 included the T-RFs of 91 bp and 508 bp, which might be closely related to *Candidatus Methylospira*. Both cluster 2 and cluster 3 could be affiliated with Type Ib MOB, but they distinctly differed in phylogeny and morphology (Danilova et al., 2016). Cluster 4 comprised of the T-RFs of 217, 370, and 403 bp, and it was phylogenetically related to Type IIa MOB (*Methylocystaceae* in *Alphaproteobacteria*). The 403 bp T-RF was likely affiliated with *Methylosinus*, while 217 and 370 bp T-RFs could not be convincingly assigned to a single genus.

The 508 bp fragment could be affiliated with either *Methylospira* or unknown Type Ia MOB. Considering the low abundance of 508 bp T-RF (<0.5% in DNA TRFLP profile and approximately 2% in RNA TRFLP profile), and in order to avoid incorrect annotation, this T-RF was excluded from further analysis.

### T-RFLP Diversity and Taxonomic Profiles of *pmoA* Gene and Transcripts

Diversity of each community was calculated based on T-RFLP results. In the current study, the T-RFs with relative abundance more than 5% in at least one sample or with average relative abundance more than 2% in all samples were defined as major T-RFs. For a given sample, the total number of T-RFs and the number of major T-RFs were greater in RNA T-RFLP profile than in DNA T-RFLP profile. On day 1, ammonium amended microcosms tended to present lower *pmoA* gene diversity than un-amended microcosm, while an opposite trend was found at days 7 and 14 (Table 1). For a given sample, *pmoA* transcript showed higher Shannon diversity than *pmoA* gene. Ammonium amended microcosms tended to have lower *pmoA* transcript diversity than un-amended microcosm. In the microcosms with treatments A–D (0–50 mM ammonium), *pmoA* transcript diversity tended to increase with time. However, the Shannon diversity of transcriptional T-RFs experienced an increase followed by a decrease in the microcosms with treatments E and F (100–200 mM ammonium).

**TABLE 1:** Numbers of T-RFs and T-RF-based Shannon diversity.

Sample	DNA		RNA	
		
	T-RFs	Shannon	T-RFs	Shannon
A1	11	1.55	23	2.55
B1	11	1.55	23	2.23
C1	11	1.49	22	2.17
D1	11	1.27	14	1.70
E1	11	1.48	20	2.12
F1	12	1.66	25	2.55
A7	12	1.74	28	2.92
B7	12	1.80	32	3.09
C7	13	1.77	27	2.86
D7	12	1.79	18	2.46
E7	14	1.89	30	2.98
F7	13	1.70	24	2.70
A14	13	1.70	36	3.17
B14	12	1.79	38	3.09
C14	12	1.83	33	2.96
D14	12	1.87	34	3.08
E14	12	1.83	32	2.89
F14	12	1.79	20	2.18

*For sample name, upper case letters refer to treatment while digits indicate sampling time.*

**FIGURE 4:**
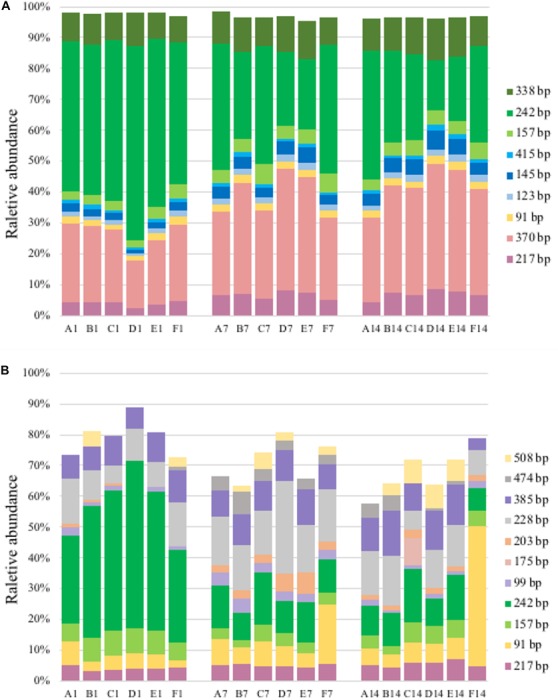
T-RFLP profiles based on *pmoA* gene **(A)** and transcripts **(B)**. For sample name, upper case letters refer to treatment while digits indicate sampling time.

A total of 11–14 T-RFs were retrieved from T-RFLP analysis of DNA samples. Most of them (including all major T-RFs) could be well assigned to certain methanotrophic groups (Figures 3, 4A). In all DNA samples, Type Ia and Type IIa methanotrophs dominated MOB communities. On day 1, the 242 bp T-RF (*Methylobacter*-related Type Ia methanotrophs) comprised about 50% of MOB communities. The 370 bp T-RF (Type IIa methanotrophs) also showed a considerable proportion (20–25%). The addition of ammonium tended to induce no considerable change of MOB community structure after 12-h incubation. After 7 and 14 days of incubation, the proportions of major T-RFs illustrated an evident variation. The proportion of Type Ia methanotrophs (157, 242, and 338 bp; marked in green) decreased with time, while Type IIa methanotrophs (217 and 370 bp, marked in pink) increased. The proportion of *Methylococcus*-related Type Ib methanotrophs (marked in blue) also increased, especially the 145 bp T-RF, whereas the proportion of *Methylospira*-related Type Ib methanotrophs (91 bp, marked in yellow) did not show a notable variation.

A total of 14–38 T-RFs were retrieved from T-RFLP analysis of RNA samples, but most of them were only detected in a few samples with low relative abundance (Figure 4B). Among the major transcript T-RFs, only 4 transcript T-RFs could be assigned to a known methanotrophic group, and on day 1 they comprised of a considerable part of MOB community in un-amended microcosm (43–72%) and of in amended microcosms (22–72%), while the other 7 T-RFs were not found in *pmoA* gene library as well as DNA T-RFLP profiles. Compared with *pmoA* gene, the community structure of *pmoA* transcripts was more sensitive to external ammonium addition. The addition of ammonium induced a marked shift in *pmoA* transcriptional community structure after 12-h incubation. The proportion of 242 bp increased, but the proportion of 91 bp decreased. After 1 or 2 weeks’ incubation, the microcosms with treatments B, C, D and E (5–100 mM ammonium) had similar transcriptional community structure as the un-amended microcosm. However, the microcosm with treatment F (with the highest ammonium addition) encountered a remarkable increase in 91 bp (Ca. *Methylospira*-related Type Ib methanotrophs). Moreover, the 370 bp T-RF, accounting for up to one fourth (average) of DNA T-RFs, was only detected on day 14, with relative abundance of 0.8–2.9%.

### Clustering and Statistical Analysis of MOB Communities

**FIGURE 5:**
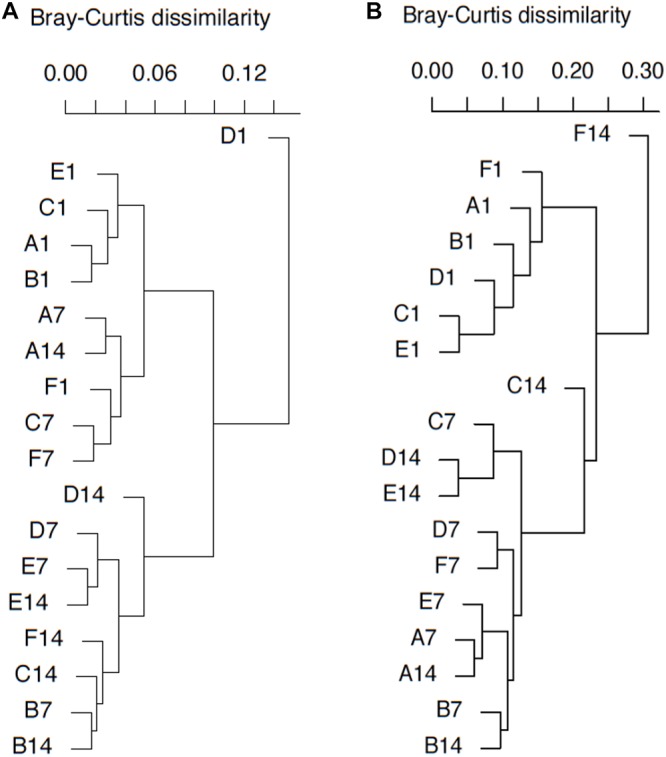
*pmoA* gene **(A)** and transcripts **(B)**-based cluster diagrams of similarity values for samples with different treatments. Dissimilarity levels are indicated above the diagram. For sample name, upper case letters refer to treatment while digits indicate sampling time.

DNA- and RNA-based MOB community structures were characterized with hierarchal clustering based on Bray–Curtis dissimilarity (Figure 5). *pmoA* community structure was quite stable during the whole incubation period. Most of the samples on day 1 were grouped together. Samples B7, D7, E7, B14, C14, D14, E14, and F14 were clustered into another group. Sample D1 was distantly separated from other samples. Higher dissimilarity of transcriptional community structures could be observed among samples. The samples on day 1 were still close to each other, and they were clearly separated from the samples at day 7 and 14. Samples A7, A14, B7, B14, D7, E7, and F7 could form a clade, while samples C7, D14, and E14 formed another clade. Moreover, sample F14 was distantly separated from other samples.

RDA with permutation test was carried out to test the potential relationship between T-RF-based composition and influential factors (treatment and incubation time). The result indicated that incubation time had a significant impact on DNA-based MOB community composition (*P* < 0.01), while ammonium concentration did not exert a significant influence (*P* > 0.05). The constrained variables could explain up to 74.4% of total variance. However, most of the explained variance (73.7% out of 74.4%) was related to constrained axis 1, and only the first axis was significant (*P* = 0.029). For RNA-based MOB community, treatment and time together explained 76.0% of total variance. These results indicated that after the addition and with the depletion of ammonium, the community compositions of both *pmoA* gene and transcripts could undergo a considerable shift.

## Discussion

### Effect of Ammonium on MOP

The current study showed that a high dosage of ammonium could present a temporary inhibition effect on methane oxidation. The result was consistent with several previous studies carried out in various ecosystems (Bosse et al., 1993; Nold et al., 1999; Murase and Sugimoto, 2005). These studies indicated that the addition of ammonium might inhibit methane oxidation in water and sediment of freshwater lakes. However, previous studies conflicted on the influence of ammonium on methane oxidation in lake sediment. 4 mM of ammonium in pore water was reported to inhibit methane oxidation in Lake Constance (Bosse et al., 1993), whereas a continuous water flow containing up to 15 mM of ammonium did not obviously affect methane oxidation in Lake Kevätön (Liikanen and Martikainen, 2003).

In the present study, inhibition was only observed in the microcosm with a very high ammonium dosage (with 17.3 mM ammonium in overlying water on day 1), while no evident inhibition was found in the other ammonium amended microcosms, even at high dosages. This suggested that methane oxidation could be significantly inhibited by excess ammonium addition, which was also supported by the result of two-way ANOVA. However, there might be a minimal concentration for ammonium to present the inhibition effect, and this threshold varied among different lakes. In Dianchi Lake, where the ammonium nitrogen (NH_4_^+^-N) concentration of pore water was 344 μM, a much higher ammonium concentration was needed to inhibit methane oxidation than that in the oligotrophic Lake Constance (Bosse et al., 1993). While in the hyper-eutrophic Lake Kevätön, the inhibition threshold was similar to that in Dianchi Lake. Therefore, the threshold might be related to *in situ* ammonium concentration. During the long-term eutrophication, *in situ* methanotrophs were adapted to high concentration of ammonium nitrogen. And the adaption of microbial community might be a major contributor to the difference in inhibition threshold.

Despite of a very high dosage of ammonium in group F, sediment MOP was only partially inhibited. This might be explained by two facts. The studied sediment sample originated from a eutrophic lake, which suffered from high nitrogen input. Methanotrophs in this kind of ecosystem could effectively oxidize methane under the condition of high ammonium concentration, as is also observed in Lake Kevätön (Liikanen and Martikainen, 2003). This was consistent with the above-mentioned lake-related minimal inhibition concentration for methane oxidation. On the other hand, the affinity of pMMO (*pmoA* encoding protein) to methane is much higher than that to ammonium (Bédard and Knowles, 1989). As a result, when the methane concentration is high enough, as a common case for the measurement of MOP, methanotrophs were still able to consume a considerable amount of methane.

In this study, after the depletion of ammonium, sediment MOP could also get a quick recovery. The highest ammonium dosage eventually stimulated sediment MOP in the long run (about 2 weeks). A similar recovery of MOP after a single-shot fertilization has also been reported in forest soil (Borjesson and Nohrstedt, 2000). In the current study, the shifts in functional gene density, composition, and transcription were observed after the 2 weeks’ incubation. Considering the increase of *pmoA* gene abundance and the change of RNA-based MOB community structure, the recovery of MOP might be attributed to an adaption to the environment.

### Effect of Ammonium on *pmoA* Gene and Transcript Abundance

So far, little is known about the changes of MOB abundance and transcripts induced by external ammonium amendment in lake sediments. Yet a few studies have addressed DNA- and RNA-level shifts in other ecosystems. Alam and Jia (2012) reported that the addition of 200 μg of nitrogen/g dry weight soil (in ammonium sulfate) showed no significant influence on *pmoA* gene abundance in paddy soil. However, in ammonium-amended rhizospheric soil microcosms, *pmoA* gene abundance slightly increased after 29 days’ incubation (Shrestha et al., 2010). In this study, after 7 days’ incubation, the sediment microcosm with the highest ammonium dosage had much higher *pmoA* gene abundance than un-amended microcosm and other amended microcosms with lower dosage, whereas no significant difference of *pmoA* gene abundance was detected between un-amended microcosm and amended microcosms (except for the treatment with the highest ammonium dosage). After 14 days’ incubation, the microcosm with the highest ammonium dosage also had much higher *pmoA* gene-based MOB abundance than other amended microcosms. Therefore, a single shot of ammonium amendment could present a lagged and prolonged effect. The change of community density could still be observed even after the depletion of ammonium. Considering the above-mentioned studies, there might also be an ammonium concentration threshold to induce community shift. Dianchi Lake had been suffering from eutrophication for over 30 years (Huang et al., 2017). It could be assumed that MOB community in this lake had been adapted to high *in situ* ammonium concentration. As a result, only extremely high dosage of ammonium could pose a significant impact on DNA-based MOB abundance. However, most studies relevant to methanotroph density and transcription were based on soil ecosystem. Works are still needed to detect the response pattern of methanotrophs to ammonium amendment in freshwater lake.

At each time point, the microcosm with no external ammonium addition had lower abundance of *pmoA* transcripts than each amended microcosm. This suggested the transcription of *pmoA* gene was induced by the addition of ammonium. And it is obvious that transcription was more sensitive than functional gene abundance. The shift of number of transcripts was observed soon after ammonium amendment (12 h). And after the depletion of ammonium (14 days), the number of transcripts showed no significant difference. The decoupled variation of MOP and *pmoA* gene transcription might be a key factor to explain the inter-lake difference in minimal inhibition concentration. In eutrophic lakes, the active methanogenesis and the consistent high concentration of ammonium maintained an abundant and active methanotrophic community (Liu et al., 2015). When amended with a moderate dosage of ammonium, the shift in *pmoA* gene transcription was sufficient to make up for the competition between methane and ammonium for the binding site of pMMO (Bédard and Knowles, 1989). As a result, there would not be a notable decrease in MOP. Therefore, the sensitive response of *in situ* methanotroph transcription to ammonium amendment might be the major contributor to the epigenetic minimal inhibition concentration.

### Effect of Ammonium on DNA- and RNA-Based Methanotrophic Community Compositions

Several previous studies have investigated the influence of ammonium amendment on soil MOB community structure (Mohanty et al., 2006; Shrestha et al., 2010; Alam and Jia, 2012), yet information about the influence of ammonium amendment on freshwater MOB community structure and functional gene transcription is still lacking. In the current study, DNA-based T-RF profiling changed with incubation time, but ammonium concentration did not present a significant impact. This could be partially explained by the short incubation time compared with microbial growth rate (Borrel et al., 2011). The ammonium concentration, lower than or close to the minimal inhibition concentration, decreased to *in situ* level shortly after amendment. Therefore, the residual ammonium may not be sufficient to induce a significant community shift in such a short period.

*pmoA* mRNA was much more sensitive to ammonium amendment. Immediately after ammonium addition (after 12-h incubation), the relative abundance of Type I (especially Type Ia) methanotrophs transcripts increased, instead of Type II. This coincided with the result reported in rice and forest soils (Mohanty et al., 2006). This also suggested that a high level of ammonium favored the growth of Type I methantrophs and they might play an important role in methane oxidation in ammonium-rich lake. However, both DNA- and RNA-based T-RFLP profiles indicated that the addition of ammonium lead to an increase in the ratio of Type II to Type I methanotrophs in 2 weeks, which was contrary to the results observed in some previous studies in soil ecosystems (Bodelier et al., 2000; Mohanty et al., 2006; Alam and Jia, 2012). These previous studies found that Type I methanortrophs had a numerical advantage over Type II at a high ammonium concentration. Our recent field study suggested that that the abundance of Type II methanotrophs in sediment of Dianchi Lake was closely correlated to the concentration of ammonium (Yang et al., 2016). Although the Type I and Type II methanotrophs were affiliated with different classes, metabolic and adaptive characteristics might not be consisted with this classification. And the dominant genus varied greatly among different type of ecosystems (Borrel et al., 2011). Therefore, the response of methanotrophs to ammonium might largely depend on the characteristics of ecosystem. In addition, the community compositions of *pmoA* genes and transcripts could be divergent, and the composition of *pmoA* genes and *pmoA* transcripts could show different responses to ammonium addition.

## Conclusion

This was the first microcosm study addressing the influence of ammonium on freshwater lake sediment MOB community and functional gene transcription. The MOB community density and transcription were both stimulated by ammonium amendment, whereas net methane oxidation could be partially inhibited at a very high ammonium concentration. During 2 weeks’ incubation, MOB community composition did not show a significant shift after ammonium amendment. However, the composition of *pmoA* gene transcripts was more sensitive to ammonium.

## Data Availability Statement

The datasets generated for this study can be found in the GenBank MH189486–MH189506.

## Author Contributions

SX and YL conceived and designed the experiments. YY, TT, and JC performed the experiments. YY analyzed the data and wrote the manuscript. SX revised the manuscript.

## Conflict of Interest

The authors declare that the research was conducted in the absence of any commercial or financial relationships that could be construed as a potential conflict of interest.

## Footnotes

1 http://www.restrictionmapper.org

## References

[B1] Alam M. Jia Z. (2012). Inhibition of methane oxidation by nitrogenous fertilizers in a paddy soil. *Front. Microbiol.* 3:246. 10.3389/fmicb.2012.00246 22783249 PMC3389332

[B2] Bastviken D. Cole J. Pace M. de Bogert M. (2008). Fates of methane from different lake habitats: connecting whole-lake budgets and CH4 emissions. *J. Geophys. Res. Biogeosci.* 113:G02024.

[B3] Bastviken D. Cole J. Pace M. Tranvik L. (2004). Methane emissions from lakes: dependence of lake characteristics, two regional assessments, and a global estimate. *Glob. Biogeochem. Cycle* 18: GB4009.

[B4] Bédard C. Knowles R. (1989). Physiology, biochemistry, and specific inhibitors of CH4, NH4+, and CO oxidation by methanotrophs and nitrifiers. *Microbiol. Rev.* 53 68–84. 10.1128/mmbr.53.1.68-84.1989 2496288 PMC372717

[B5] Bodelier P. L. E. Laanbroek H. J. (2004). Nitrogen as a regulatory factor of methane oxidation in soils and sediments. *FEMS Microbiol. Ecol.* 47 265–277. 10.1016/S0168-6496(03)00304-0 19712315

[B6] Bodelier P. L. E. Roslev P. Henckel T. Frenzel P. (2000). Stimulation by ammonium-based fertilizers of methane oxidation in soil around rice roots. *Nature* 403 421–424. 10.1038/35000193 10667792

[B7] Borjesson G. Nohrstedt H. O. (2000). Fast recovery of atmospheric methane consumption in a Swedish forest soil after single-shot N-fertilization. *For. Ecol. Manage.* 134 83–88. 10.1016/s0378-1127(99)00249-2

[B8] Borrel G. Jezequel D. Biderre-Petit C. Morel-Desrosiers N. Morel J. P. Peyret P. (2011). Production and consumption of methane in freshwater lake ecosystems. *Res. Microbiol.* 162 832–847. 10.1016/j.resmic.2011.06.004 21704700

[B9] Bosse U. Frenzel P. Conrad R. (1993). Inhibition of methane oxidation by ammonium in the surface layer of a littoral sediment. *FEMS Microbiol. Ecol.* 13 123–134. 10.1111/j.1574-6941.1993.tb00058.x 16535750

[B10] Dam B. Dam S. Kim Y. Liesack W. (2014). Ammonium induces differential expression of methane and nitrogen metabolism-related genes in Methylocystis sp strain SC2. *Environ. Microbiol.* 16 3115–3127. 10.1111/1462-2920.12367 24373058

[B11] Danilova O. V. Suzina N. E. Van De Kamp J. Svenning M. M. Bodrossy L. Dedysh S. N. (2016). A new cell morphotype among methane oxidizers, a spiral-shaped obligately microaerophilic methanotroph from northern low-oxygen environments. *ISME J.* 10 2734–2743. 10.1038/ismej.2016.48 27058508 PMC5113839

[B12] Fergala A. AlSayed A. Eldyasti A. (2018). Behavior of type II methanotrophic bacteria enriched from activated sludge process while utilizing ammonium as a nitrogen source. *Int. Biodeterior. Biodegrad.* 130 8–16. 10.1016/j.ibiod.2018.03.010

[B13] Huang C. C. Yao L. Zhang Y. L. Huang T. Zhang M. L. Zhu A. X. (2017). Spatial and temporal variation in autochthonous and allochthonous contributors to increased organic carbon and nitrogen burial in a plateau lake. *Sci. Total Environ.* 603 390–400. 10.1016/j.scitotenv.2017.06.118 28633116

[B14] Jang I. Lee S. Zoh K. D. Kang H. (2011). Methane concentrations and methanotrophic community structure influence the response of soil methane oxidation to nitrogen content in a temperate forest. *Soil Biol. Biochem.* 43 620–627. 10.1016/j.soilbio.2010.11.032

[B15] Kumar S. Stecher G. Tamura K. (2016). MEGA7: molecular evolutionary genetics analysis version 7.0 for bigger datasets. *Mol. Biol. Evol.* 33 1870–1874. 10.1093/molbev/msw054 27004904 PMC8210823

[B16] Letunic I. Bork P. (2016). Interactive tree of life (iTOL) v3, an online tool for the display and annotation of phylogenetic and other trees. *Nucleic Acids Res.* 44 W242–W245. 10.1093/nar/gkw290 27095192 PMC4987883

[B17] Liikanen A. Martikainen P. J. (2003). Effect of ammonium and oxygen on methane and nitrous oxide fluxes across sediment–water interface in a eutrophic lake. *Chemosphere* 52 1287–1293. 10.1016/s0045-6535(03)00224-8 12852980

[B18] Liu Y. Zhang J. X. Zhao L. Li Y. Z. Yang Y. Y. Xie S. G. (2015). Aerobic and nitrite-dependent methane-oxidizing microorganisms in sediments of freshwater lakes on the Yunnan Plateau. *Appl. Microbiol. Biotechnol.* 99 2371–2381. 10.1007/s00253-014-6141-5 25698510

[B19] Mohanty S. R. Bodelier P. L. Floris V. Conrad R. (2006). Differential effects of nitrogenous fertilizers on methane-consuming microbes in rice field and forest soils. *Appl. Environ. Microbiol.* 72 1346–1354. 10.1128/aem.72.2.1346-1354.2006 16461686 PMC1392950

[B20] Murase J. Sugimoto A. (2005). Inhibitory effect of light on methane oxidation in the pelagic water column of a mesotrophic lake (Lake Biwa, Japan). *Limnol. Oceanogr.* 50 1339–1343. 10.4319/lo.2005.50.4.1339

[B21] Nold S. C. Boschker H. T. S. Pel R. Laanbroek H. J. (1999). Ammonium addition inhibits 13C-methane incorporation into methanotroph membrane lipids in a freshwater sediment. *FEMS Microbiol. Ecol.* 29 81–89. 10.1111/j.1574-6941.1999.tb00600.x

[B22] Nyerges G. Stein L. Y. (2009). Ammonia cometabolism and product inhibition vary considerably among species of methanotrophic bacteria. *FEMS Microbiol. Lett.* 297 131–136. 10.1111/j.1574-6968.2009.01674.x 19566684

[B23] Oksanen J. Blanchet F. G. Friendly M. Kindt R. Legendre P. McGlinn D. (2018). *Vegan, Community Ecology Package, Version 2.4-6.*

[B24] Rudd J. W. M. Furutani A. Flett R. J. Hamilton R. D. (1976). Factors controlling methane oxidation in Shield Lakes: the role of nitrogen fixation and oxygen concentration. *Limnol. Oceanogr.* 21 357–364. 10.4319/lo.1976.21.3.0357

[B25] Seghers D. Top E. M. Reheul D. Bulcke R. Boeckx P. Verstraete W. (2003). Long-term effects of mineral versus organic fertilizers on activity and structure of the methanotrophic community in agricultural soils. *Environ. Microbiol.* 5 867–877. 10.1046/j.1462-2920.2003.00477.x 14510840

[B26] Shrestha M. Shrestha P. M. Frenzel P. Conrad R. (2010). Effect of nitrogen fertilization on methane oxidation, abundance, community structure, and gene expression of methanotrophs in the rice rhizosphere. *ISME J.* 4 1545–1556. 10.1038/ismej.2010.89 20596069

[B27] Veraart A. J. Steenbergh A. K. Ho A. Kim S. Y. Bodelier P. L. E. (2015). Beyond nitrogen: the importance of phosphorus for CH4 oxidation in soils and sediments. *Geoderma* 259 337–346. 10.1016/j.geoderma.2015.03.025

[B28] Wang S. H. (2012). *Manual for Sediment Mass Investigation and Assessment.* Beijing: Science Press.

[B29] Yang Y. Y. Zhao Q. Cui Y. H. Wang Y. L. Xie S. G. Liu Y. (2016). Spatio-temporal variation of sediment methanotrophic microorganisms in a large eutrophic lake. *Microb. Ecol.* 71 9–17. 10.1007/s00248-015-0667-7 26318324

[B30] Zhang X. Kong J. Y. Xia F. F. Su Y. He R. (2014). Effects of ammonium on the activity and community of methanotrophs in landfill biocover soils. *Syst. Appl. Microbiol.* 37 296–304. 10.1016/j.syapm.2014.03.003 24794017

