# Supplementary material for: Ammonium Impacts Methane Oxidation and Methanotrophic Community in Freshwater Sediment
Source: Front Bioeng Biotechnol. 2020 Mar 31;8:250. doi: 10.3389/fbioe.2020.00250 (PMC7137091; doi:10.3389/fbioe.2020.00250)

**Supplementary Material**

**Journal name：Frontiers in Bioengineering and Biotechnology**

**Article title:** Ammonium impacts methane oxidation and methanotrophic community in freshwater sediment

**Author names:** Yuyin Yang^1^, Tianli Tong^1^, Jianfei Chen^1^, Yong Liu^2,*^, Shuguang Xie^1,*^

^1^State Key Joint Laboratory of Environmental Simulation and Pollution Control, College of Environmental Sciences and Engineering, Peking University, Beijing 100871, China

^2^Key Laboratory of Water and Sediment Sciences (Ministry of Education), College of Environmental Sciences and Engineering, Peking University, Beijing 100871, China

**Corresponding author.**  Tel: 86-10-62758599. Fax: 86-10-62758599.

Email: yongliu@pku.edu.cn (Y. Liu); xiesg@pku.edu.cn (S. Xie)

**Methods**

In pilot experiments, the widely used enzyme *Msp*I for T-RFLP anaylsis of *pmoA* gene could not perform well with our samples. Very few T-RFs was generated, making it hard to demonstrate MOB community diversity. Therefore, we applied in-silico analysis to determine which enzyme was more suitable for our samples. A previous NGS result (under accession number SRP131884) of methanotrophic community in the same geographic region was used as a reference. The *pmoA* sequences were grouped into OTUs at 0.03 cutoff, and the representative sequences and relative abundance of each OTU were used in further analysis. We tested all the enzymes listed in <http://www.restrictionmapper.org>. For each enzyme, we predicted the length of T-RF it generated with each representative sequence, and calculated the proportion of each different length of T-RF, resulting in a predicted T-RF profiling with each enzyme on our test dataset. A simplified T-RF map (including the enzymes which could cut over 80% of total sequences) was shown in Fig S4.

**Discussion**

T-RFLP has been a popular approach to capture microbial community diversity. It has been also widely used in community studies of methanotrophs (Mohanty et al. 2006; Pester et al. 2004; Shrestha et al. 2010). Compared with NGS (next generation sequencing), T-RLFP analysis requires much less time and sampling preparation, making it an efficient tool for monitoring community shift at short time intervals. However, the most widely used digestive enzyme, *Msp*I, could not perform satisfactorily for all kinds of samples. It either resulted in only a few T-RFs, or could not efficiently tell between different taxon. For example, only a few T-RFs were retrieved from samples from littoral Lake Constance. And most of the clones from Type I were at the same T-RF length of 248 bp (Pester et al. 2004). *Msp*I enzyme also generated very few T-RFs for the sediment samples from Dianchi Lake. The number of T-RFs based on a certain digestive enzyme might partly depend on the in-situ microbial community features. Therefore, it is of great importance to choose enzyme according to sampling source.

Since T-RF was only related to the first cleavage site, if a specific point mutation got widespread (especially when the population was limited), it might notably impact the T-RF pattern. Efforts were made to avoid some of the weakness of T-RFLP and improve the taxonomic resolution, including the application of multiplex T-RFLP (Elliott et al. 2012) and the usage of primers labelled with different fluorochromes (Deutzmann et al. 2011). Here, we tried to choose a suitable enzyme based on in-silico analysis. We expected that a suitable digestive enzyme should: (1) generate no or few T-RFs smaller than 50 bp or larger than 500 bp; (2) generate T-RFs having at least 2 bp differences among each other; (3) generate more T-RFs to retrieve the diversity; (4) be consistent with taxon and phylogenetic tree (i.e., the same T-RF should not be affiliated to very distantly related taxa). Based on these rules, most of the enzymes were easy to exclude. *Msp*I and *BciT130*I, which shared the same cleavage sites with *Hpa*II and *EcoR*II, respectively, were among the enzymes performed best with rules (1)-(3). And they were further tested based on a neighbor-joining tree. *BciT130*I had a better taxonomic resolution than *Msp*I did. Several subgroups of Type I methanotrophs could be well distinguished. It was able to generate more different T-RFs, and thus could more ideally reflect methanotrophic diversity. Therefore, we considered the *Msp*I enzyme most suitable for samples from the studied lakes. The in-silico prediction of TRFLP digestion was also proven to be an effective approach to choose proper digestive enzyme.

**References**

Deutzmann, J. S. S., Wörner, S. and Schink, B., 2011. Activity and diversity of methanotrophic bacteria at methane seeps in eastern Lake Constance sediments, Appl. Environ. Microbiol. 77, 2573-2581.

Elliott, G., Thomas, N., MacRae, M., Campbell, C., Ogden, I., Singh, B., 2012. Multiplex T-RFLP Allows for increased target number and specificity: Detection of *Salmonella enterica* and six species of *Listeria* in a single test. Plos One, 7, e43672.

Mohanty, S.R., Bodelier, P.L., Floris, V., Conrad, R., 2006. Differential effects of nitrogenous fertilizers on methane-consuming microbes in rice field and forest soils. Appl. Environ. Microbiol. 72, 1346–1354.

Pester, M., Friedrich, M., Schink, B., Brune, A., 2004. *pmoA*-based analysis of methanotrophs in a littoral lake sediment reveals a diverse and stable community in a dynamic environment, Appl. Environ. Microbiol. 70, 3138-3142.

[Shrestha, M](http://apps.webofknowledge.com/DaisyOneClickSearch.do?product=WOS&search_mode=DaisyOneClickSearch&colName=WOS&SID=8CPhC4vbj1daMb534yc&author_name=Shrestha,%20M&dais_id=4569386&excludeEventConfig=ExcludeIfFromFullRecPage)., [Shrestha, P.M](http://apps.webofknowledge.com/DaisyOneClickSearch.do?product=WOS&search_mode=DaisyOneClickSearch&colName=WOS&SID=8CPhC4vbj1daMb534yc&author_name=Shrestha,%20PM&dais_id=3336831&excludeEventConfig=ExcludeIfFromFullRecPage)., [Frenzel, P](http://apps.webofknowledge.com/DaisyOneClickSearch.do?product=WOS&search_mode=DaisyOneClickSearch&colName=WOS&SID=8CPhC4vbj1daMb534yc&author_name=Frenzel,%20P&dais_id=769472&excludeEventConfig=ExcludeIfFromFullRecPage)., [Conrad, R](http://apps.webofknowledge.com/DaisyOneClickSearch.do?product=WOS&search_mode=DaisyOneClickSearch&colName=WOS&SID=8CPhC4vbj1daMb534yc&author_name=Conrad,%20R&dais_id=41654&excludeEventConfig=ExcludeIfFromFullRecPage)., 2010. Effect of nitrogen fertilization on methane oxidation, abundance, community structure, and gene expression of methanotrophs in the rice rhizosphere. ISME J 4, 1545–1556.

**Table S1** Physicochemical parameters of sediment samples.

| TOC  (g/kg) | TN  (g/kg) | TOC/TN | NO_3_^−^-N  (mg/kg) | NH_4_^+^-N  (mg/kg) | TP  (g/kg) | pH |
| --- | --- | --- | --- | --- | --- | --- |
| 41.3 | 3.95 | 10.5 | 12.3 | 364 | 0.60 | 7.2 |

**Table S2** Multiple comparison of treatments by means of Student-Newman-Keuls post-hoc testing. In each column, different characters indicate significant difference at 0.05 level.

| treatment | MOP | | *pmoA* abundance | | transcript abundance | |
| --- | --- | --- | --- | --- | --- | --- |
|  | Day 1 | Day 14 | Day 7 | Day 14 | Day 1 | Day 7 |
| A | a | b | b | ab | d | b |
| B | a | ab | b | b | c | b |
| C | a | b | b | b | a | b |
| D | a | b | b | b | b | b |
| E | a | ab | b | b | b | b |
| F | b | a | a | a | b | a |

**Fig. S1.** Geographic location of sampling site

**
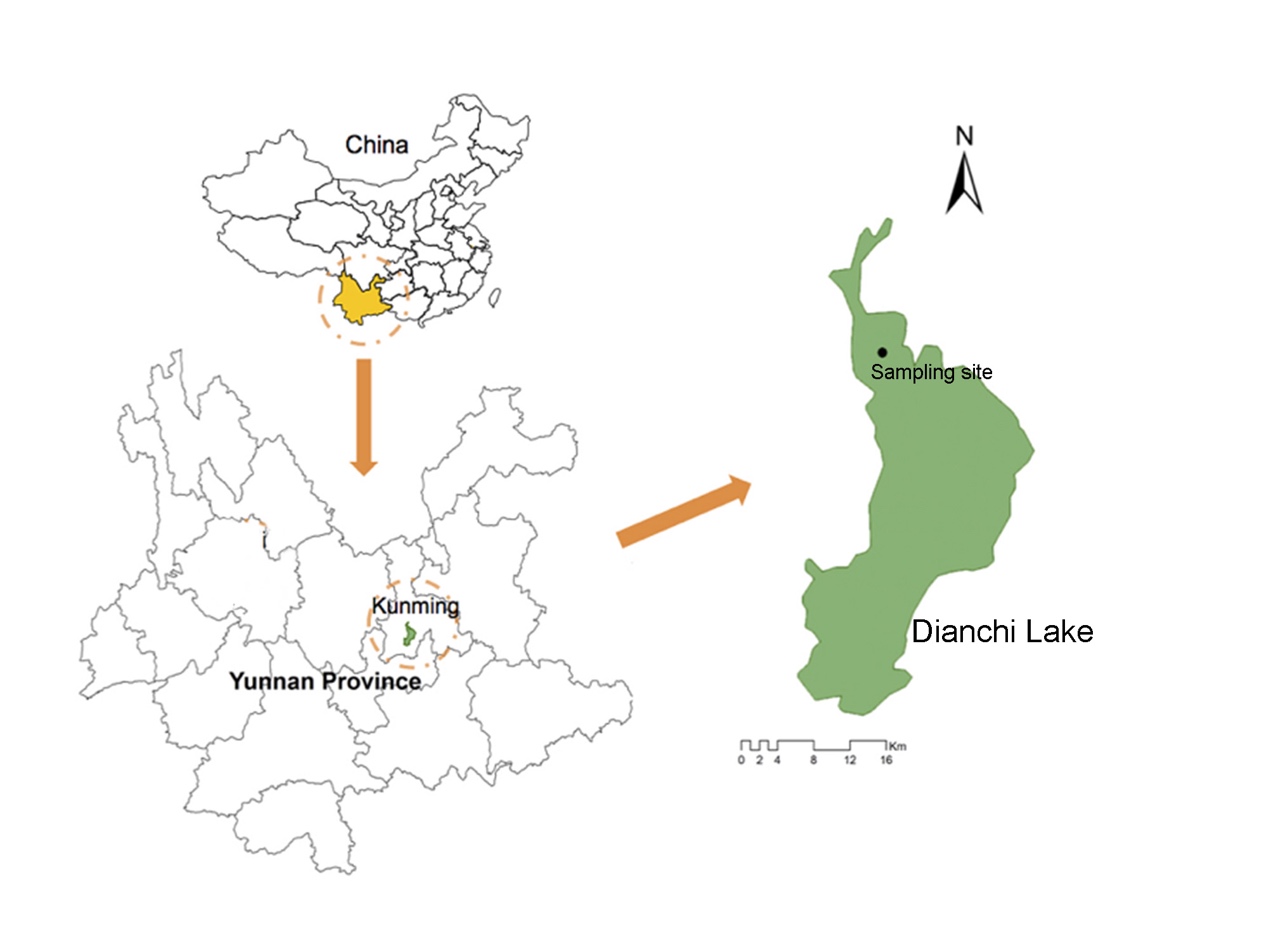
**

**Fig. S2**. Change of ammonium concentration of pore water in the microcosms with different treatments


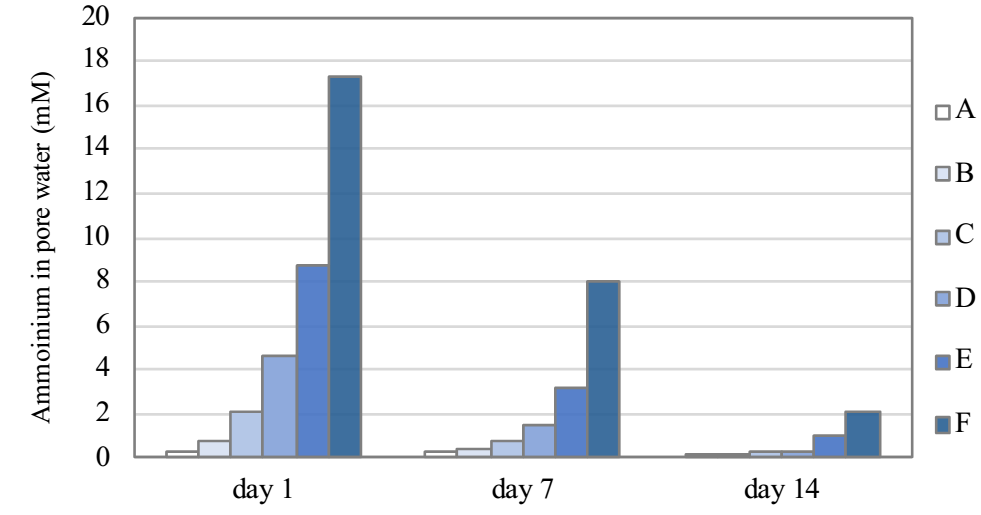


**Fig. S3.** The ratio of transcripts to *pmoA* gene in the microcosms with different treatments


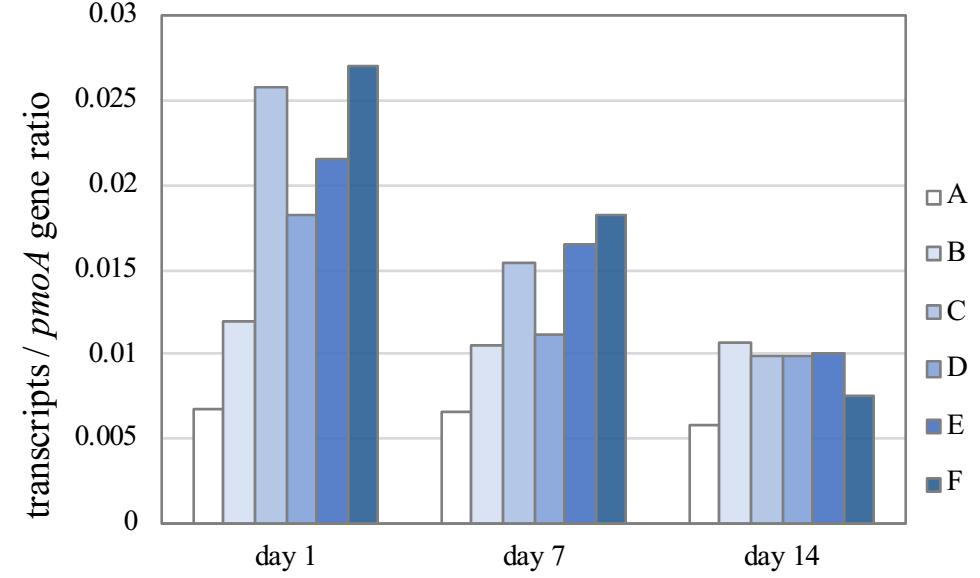


**Fig. S4.** Prediction of NGS representative sequences based on T-RFs. Sequencing data was from a previous study considering the same area, and was deposited in GenBank database under accession number SRP131884.


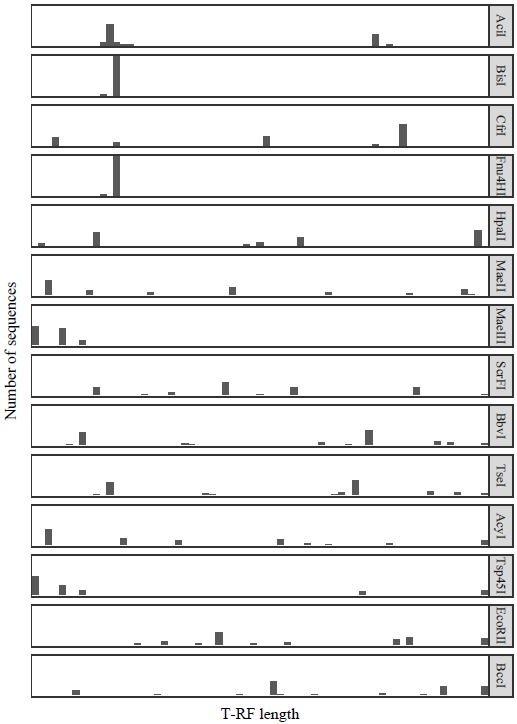

Supplement: Supplementary file 1 [file Table_1.DOCX]
